# Effects of culling vampire bats on the spatial spread and spillover of rabies virus

**DOI:** 10.1126/sciadv.add7437

**Published:** 2023-03-10

**Authors:** Mafalda Viana, Julio A. Benavides, Alice Broos, Darcy Ibañez Loayza, Ruby Niño, Jordan Bone, Ana da Silva Filipe, Richard Orton, William Valderrama Bazan, Jason Matthiopoulos, Daniel G. Streicker

**Affiliations:** ^1^ School of Biodiversity, One Health and Veterinary Medicine, College of Medical, Veterinary and Life Sciences, University of Glasgow, Glasgow G12 8QQ, UK.; ^2^ MIVEGEC, IRD, CNRS, Université de Montpellier, Montpellier, France.; ^3^ Doctorado en Medicina de la Conservación y Centro de Investigación para la Sustentabilidad, Facultad de Ciencias de la Vida, Universidad Andrés Bello, República 440 Santiago, Chile.; ^4^ MRC-University of Glasgow Centre for Virus Research, Glasgow G61 1QH, UK.; ^5^ Gobierno Regional de Apurímac, Abancay, Perú.; ^6^ Colegio Médico Veterinario de Apurímac, Abancay, Perú.; ^7^ ILLARIY (Asociación para el Desarrollo y Conservación de los Recursos Naturales), Lima, Perú.; ^8^ Universidad Peruana Cayetano Heredia, Lima, Perú.

## Abstract

Controlling pathogen circulation in wildlife reservoirs is notoriously challenging. In Latin America, vampire bats have been culled for decades in hopes of mitigating lethal rabies infections in humans and livestock. Whether culls reduce or exacerbate rabies transmission remains controversial. Using Bayesian state-space models, we show that a 2-year, spatially extensive bat cull in an area of exceptional rabies incidence in Peru failed to reduce spillover to livestock, despite reducing bat population density. Viral whole genome sequencing and phylogeographic analyses further demonstrated that culling before virus arrival slowed viral spatial spread, but reactive culling accelerated spread, suggesting that culling-induced changes in bat dispersal promoted viral invasions. Our findings question the core assumptions of density-dependent transmission and localized viral maintenance that underlie culling bats as a rabies prevention strategy and provide an epidemiological and evolutionary framework to understand the outcomes of interventions in complex wildlife disease systems.

## Teaser

Culling vampire bats fails to reduce rabies spillover to livestock and can stall or exacerbate viral spatial spread.

## INTRODUCTION

Reduction of wildlife population sizes via the lethal removal of animals (hereafter “culling”) is a common approach to prevent the spillover of pathogens into human or domestic animal populations (*1*). The principle behind culling is that lower densities of susceptible hosts should reduce the incidence of infection in the reservoir and, consequently, the risk of transmission to other species. At the extreme, reservoir populations might be reduced to a size threshold that triggers pathogen extinction (*2*). While culls have contributed to effective management of some host-pathogen systems such as tuberculosis in New Zealand brushtail possums, their efficacy in other systems has been undermined by unappreciated ecological complexity (*3*). Transmission modes that do not depend strictly on host density; asynchronous, spatially structured pathogen persistence; and behavioral and demographic responses among survivors of culls can render culling ineffective or counterproductive (*4*–*6*). For example, efforts to reduce bovine tuberculosis in cattle by culling badgers are thought to have boosted rather than depressed pathogen transmission due to greater mixing among survivors (*7*).

Understanding why culls succeed or fail in reducing disease transmission is central to improving efficacy or incentivizing investments in alternatives for disease control such as vaccination or reproductive suppression. However, with rare exceptions, wildlife culls are conducted for management, not scientific inquiry (*8*, *9*). Consequently, culls are typically carried out on large spatial and temporal scales but lack the controls or experimentally allocated variation in culling intensity that would facilitate distinguishing putative benefits or costs of culls from natural variation in disease incidence. Moreover, culls can comprise a single component of multifaceted management plans. Co-occurring interventions, such as vaccination of humans and domestic animals or food provisioning of wildlife reservoirs, can obscure how culling affects cross-species transmission risk or transmission dynamics in the reservoir (*10*). Last, data collected alongside culls are generally restricted to incidence of infection. The growing capacity for pathogen genomic sequencing could provide direct insights into the spatiotemporal responses of hosts and pathogens to culls, but sequencing has rarely, if ever, been carried out alongside culls. Joint ecological and evolutionary approaches that could derive mechanistic explanations and actionable insights from what are effectively uncontrolled natural experiments would remove a bottleneck to evidence-based management.

Culling of common vampire bats (*Desmodus rotundus*) represents a key example of how uncertainty in the ecological consequences of culling limits evidence-based policy. In most of Latin America, vampire bats constitute the primary source of rabies outbreaks, causing livestock mortality associated with losses of tens of millions of dollars annually and representing a perpetual public health threat through zoonotic transmission from bats to humans during blood feeding (*11*, *12*). Although vaccination is encouraged in high-risk livestock populations and following human exposure, inconsistent uptake has sustained losses in vulnerable communities (*13*). As a supplement to vaccination of these spillover hosts, efforts to control vampire bat–transmitted rabies (VBR) since the 1970s have culled bats using anticoagulant poisons (“vampiricide”), either applied topically to bats to spread by allogrooming or applied to livestock for later consumption by bats during blood feeding (*14*). The efficacy of culling bats for rabies management remains controversial. Mathematical models using data from wild vampire bats in Peru hypothesized that reactive culling could be counterproductive if it induced bat dispersal and suggested metapopulation persistence of VBR, which, if verified, would necessitate synchronization of culls over large geographic areas (*15*, *16*). However, in relying exclusively on viral exposure data inferred from antibodies in bats, these observational studies were unable to quantify how culling affected rabies incidence in bats, rates of spillover to livestock or viral spatial spread. Another study that monitored rabies incidence in livestock after culling bats in the expected path of an advancing rabies epizootic in Argentina suggested that preventive culls (i.e., before the arrival of rabies) impeded viral spatial spread (*17*). However, the lack of replication and the small spatial and temporal scales involved made it impossible to separate potential effects of culling from other factors that could influence viral spread (e.g., landscape barriers to bat movement), incidence in bats (e.g., natural fluctuations unrelated to culling), or detection via livestock surveillance systems (e.g., higher livestock vaccination coverage reducing spillover despite unchanged viral circulation in bats). As such, whether culls reduce spillover to livestock by reducing rabid bat incidence or increase rabies spillover or spatial spread by promoting bat dispersal is unresolved.

Here, we combined epidemiological and genomic data from a synchronized and geographically expansive vampire bat cull in Peru to understand the epidemiological consequences of culling for rabies transmission within the bat reservoir and to livestock. Specifically, after confirming that culling reduced bat population size using questionnaire data describing bat attacks on livestock, we tested whether culling increased or decreased spillover to livestock using Bayesian state-space models (SSMs) that incorporated culling effort alongside other covariates describing the spatiotemporal dynamics of rabies and livestock vaccination effort. SSMs are particularly suited for this challenge because they jointly model biological and observation processes, enabling probabilistic inference of intervention efficacy, underlying natural variation, and potential confounders (*18*, *19*). We next quantified how culling affected the spatial spread of rabies within vampire bat populations using whole genome sequencing of rabies viruses and Bayesian phylogeographic analysis. By considering culling activity at the origin and destination of inferred viral dispersal events, we tested the hypothesis that reactive culling in areas of active viral circulation would enhance viral spatial spread while culling before virus arrival would decelerate invasions.

## RESULTS

**Fig. 1.**
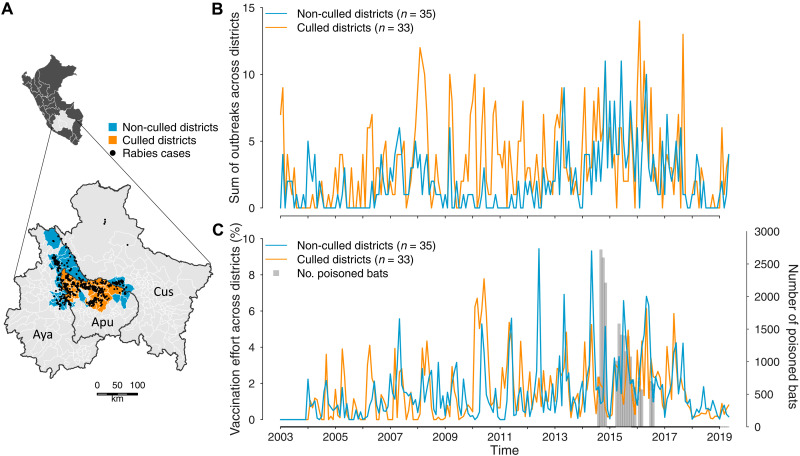
A large-scale synchronized vampire bat cull in southern Peru fails to eliminate rabies spillover. (**A**) Map of the study area in Peru and zoomed map with culled (orange) and non-culled (blue) districts within that region. Black lines indicate department borders (Aya, Ayacucho; Apu, Apurimac; Cus, Cusco), and white lines show district borders. Black points show rabies outbreaks from 2003 to 2019. Points in gray areas represent geographically isolated outbreaks that were excluded from further analyses. (**B**) Time series of livestock rabies outbreaks indicates sustained circulation of rabies in bats and spillover to livestock in the post-culling years in culled and non-culled districts. (**C**) Time series of livestock vaccination effort against rabies in culled and non-culled districts. The gray bars and the second *y* axis show culling intensity through time.

Our study area spanned three administrative regions in southern Peru [Departments of Apurimac, Ayacucho, and Cusco (AAC)], which accounted for 60.2% (annual range, 32.9 to 81.4%) of livestock rabies outbreaks nationally between 2003 and 2019 (Fig. 1, A and B). Previous phylogenetic analyses showed that a single epidemiological cycle of rabies circulates in AAC vampire bats and demonstrated the absence of alternative animal reservoirs for livestock infections (*20*). As livestock are dead end hosts for rabies, each infection represents an independent spillover event from bats, making infection patterns in livestock a reasonable proxy for rabies incidence in the bat reservoir (*13*). AAC therefore provides an unusual high-intensity spillover setting to quantify effects of culling bats on virus spillover. Although the Ministry of Agriculture of Peru culls vampire bats in all three regions sporadically in response to rabies outbreaks or farmers’ complaints of bat depredation on livestock, the Regional Government of Apurimac carried out a large geographically coordinated cull between August 2014 and August 2016, which entailed 4361 culling events across 33 districts. In total, vampiricide was applied to 21,243 bats [average, 1249 bats per month (range, 566 to 2714); average, 643 bats per district (range, 35 to 2461); Fig. 1C]. Because vampiricide spreads to up to 15 bats per individual treated, reported numbers underestimate the true number of bats killed but reflect spatial and temporal variation in culling effort (*21*, *22*). These activities represent a major supplement over standard bat control for the area. Beyond bat culling, the program included investments in livestock vaccination and education of farmers and local authorities on rabies risk and management.

**Fig. 2.**
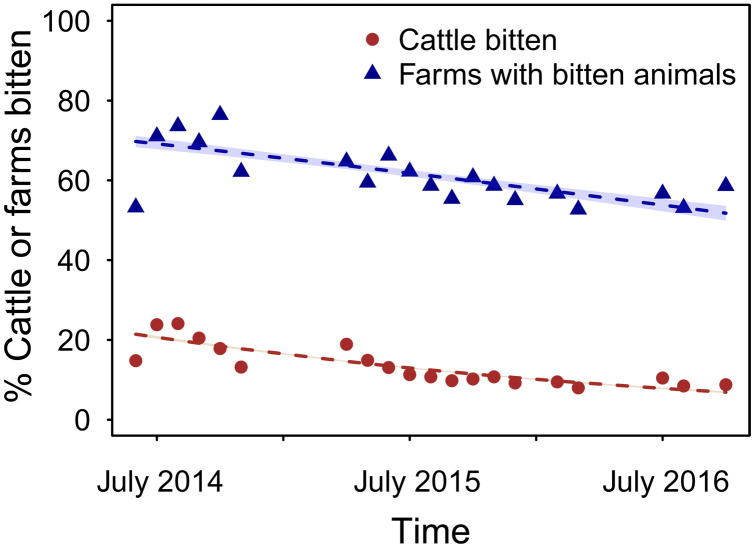
Culling reduces vampire bat abundance. Recorded monthly percent of individual animals bitten (brown circles) and of farms reporting at least one bitten animal in the previous month (blue triangles) with associated logistic regression predictions [mean dashed lines with shaded 95% confidence intervals (CIs)]. Culling took place from August 2014 until August 2016.

We first evaluated how culls affected bat population size, using the intensity of bat bites on livestock as a proxy (*23*). Specifically, we used data from questionnaires on bat bites carried out in the 33 culled districts of Apurimac (mean, 662 farms surveyed per month; range, 355 to 860). A binomial generalized linear model (GLM) and a binomial generalized linear mixed model (GLMM) showed that both the percentage of animals bitten per farm (GLMM: slope = −0.053; SD = 0.0009; *P* < 2 × 10^−16^) and the percentage of farms reporting bat bites (GLM: slope = −0.027; SD = 0.002; *P* < 2 × 10^−16^) decreased during the culling period (Fig. 2). These observations indicate that culls reduced bat population density but were insufficient to eliminate bats from most areas (Fig. 2). In the past 3 months of the culling period, 59% of farms still reported at least one bitten animal.

Having established reductions in vampire bat population size in culled districts, we next evaluated how culling affected the dynamics of rabies spillover within the context of 16 years of passive surveillance data on laboratory-confirmed livestock rabies mortality (12 years before, 2 years of culling, and 2 years after cull; total, 1029 outbreaks). The sustained occurrence of spillover in post-culling years demonstrated decisively that culls failed to eliminate rabies (Fig. 1B). However, we observed a putative decline in outbreaks that coincided with the end of the culling program in August 2016 (Fig. 1B). Because the program included educational components that would be expected to raise rabies awareness and reporting, faltering surveillance is unlikely to explain any decrease (*24*). A decline in spillover might also be observed if intensified livestock vaccination disguised unaltered levels of viral circulation in bats; however, livestock vaccination rates also declined in post-culling years (Fig. 1C). The putative decline occurred in both culled and non-culled districts and changes of similar magnitude occurred in earlier years. This implies considerable interannual variation in rabies incidence, even in the absence of large-scale coordinated culls, raising the possibility that the putative post-cull reduction of spillover could be explained by natural fluctuations in rabies incidence. Alternatively, it is conceivable that culling had geographically expansive and beneficial effects, altering spillover dynamics in both culled and non-culled districts.

**Fig. 3.**
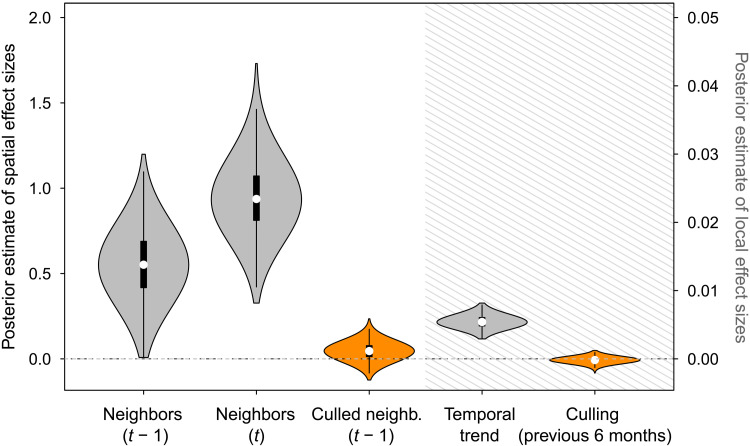
Effect sizes from the Bayesian ZIP SSM model of rabies spillover to livestock. Violins show the full posterior distribution of the effect sizes retained in the best model with internal boxplots showing the 50 and 95% quantiles. Spatial and local effects are shown in white- and gray-shaded areas, respectively. Gray violins correspond to background spatiotemporal dynamics terms, and orange violins correspond to culling terms. The effect of livestock vaccination is not shown because it was not retained by model selection.

Formally distinguishing the natural or anthropogenic mechanisms that drove the observed spatiotemporal variation in rabies incidence is vital to understand whether culling prevents rabies spillover but is complicated by uneven surveillance effort across AAC. We therefore developed a Bayesian zero-inflated Poisson SSM (ZIP SSM) that jointly described the monthly probability (i.e., occurrence) and number (i.e., intensity) of livestock rabies outbreaks per district across the 68-district study area while simultaneously modeling the observation process to account for district-specific underreporting of rabies outbreaks. The best model according to our selection criteria (see Materials and Methods) included spatial and temporal descriptors of rabies dynamics and effects of culling during the previous 6 months on both the local number of rabies outbreaks (“local culling”) and on the occurrence of rabies in neighboring districts (neighbors culling). Livestock vaccination during the previous 12 months was not retained in the most competitive models (table S1 and figs. S1 and S2). The posterior distributions of effect sizes revealed that the number of outbreaks in livestock increased over time, which may reflect a combination of geographic expansions of VBR and gradual improvements in surveillance [Fig. 3; mean trend, 0.005; 95% confidence interval (CI), 0.004 to 0.007]. The presence of rabies in neighboring districts was a major determinant of the probability of rabies outbreaks in the focal district (Fig. 3). A Royama triangle analysis of the autoregressive (AR) components of the model (i.e., on the posterior distributions of effect sizes describing rabies incidence during the preceding 1 and 2 months) supported noncyclic, enzootic maintenance of rabies across the study area (Supplementary Text and fig. S3) (*25*, *26*). Together, these results show that the model effectively captured the expected epidemiological dynamics in the AAC, characterized by viral expansions across districts and long-term maintenance by spatial processes (*15*, *27*). The model predicted time series of rabies outbreaks closely matched observed outbreaks (Supplementary Text and fig. S1). Despite being retained by model selection, the posterior distribution of effect sizes showed that local culling had no detectable impact on the number of rabies outbreaks (Fig. 3). This result may indicate that culling simultaneously increased and decreased the number of outbreaks, possibly depending on district or on whether culls were implemented after capturing bats in roosts versus foraging locations (Fig. 3; median, −0.0002; 95% CI, −0.001 to 0.0006; 75% CI, −0.0005 to 0.0002). This result shows that culls did not consistently reduce rabies spillover to livestock as intended. In contrast, neighbors culling had a marginally positive effect, with the median and 75% quantile exceeding zero (Fig. 3; mean, 0.046; 95% CI, −0.04 to 0.13; 75% CI, 0.005 to 0.08), implying that culling may have increased rather than decreased the probability of rabies outbreaks in nearby districts (Fig. 3).

To directly evaluate effects of culling on viral spatial spread, we carried out whole genome sequencing of rabies viruses collected from AAC livestock from 1997 to 2016. A preliminary phylogenetic analysis of the *nucleoprotein* gene of these viruses together with historical reference sequences showed that the majority (*n* = 306 of 320) of viruses clustered within the previously described VBR lineage 3 [L3; posterior probability (PP), 1.0; fig. S4] (*20*). An additional lineage, previously detected in northern and central Peru (hereafter L1) was also detected in 14 AAC livestock from 2004 to 2016. Most of these viruses were found at the northern limits of AAC and shared most recent common ancestors (MRCAs) with viruses collected outside of AAC, consistent with enzootic circulation of L1 in parts of Ayacucho and Cusco. More notably, four sequences detected from 2013 to 2016 formed a monophyletic clade within the core study area (PP, 1; fig. S4). The inferred timings of the MRCA of this clade and its descendant nodes within AAC were consistent with a viral incursion that entered Apurimac around June 2012 [95% Highest Posterior Density (HPD), 2012.1 to 2013.5] and spread through actively culled districts to Cusco via bat-to-bat transmission. This result suggests invasion of an additional viral lineage despite ongoing culling and the enzootic circulation of the L3 virus, each of which would be expected to deplete the susceptible bat population through mortality and natural immunization from abortive exposures (*15*, *28*).

Phylogenetic analyses of complete viral genomes from the more widespread L3 virus in Bayesian Evolutionary Analysis by Sampling Trees (BEAST) revealed two main clades that were mostly compartmentalized within culled and non-culled areas (fig. S5). This apparent epidemiological isolation corroborates the conclusion from our ZIP SSM that culling was unlikely to have reduced the intensity of spillover in non-culled districts. Continuous phylogeographic analysis showed that rabies expanded across the AAC at an average rate of 7.89 km/year [median weighted branch dispersal velocity; 95% HPD, 7.29 to 8.50; median weighted diffusion coefficient, 61.43 (95% HPD, 55.44 to 68.54); fig. S5], consistent with earlier observations (*27*). Patterns in viral effective population size inferred through the Bayesian skyride model closely resembled the number of outbreaks observed in passive surveillance data (fig. S5). Together these findings support the ability of the phylogenomic models to accurately reconstruct past viral spatial and demographic dynamics.

**Fig. 4.**
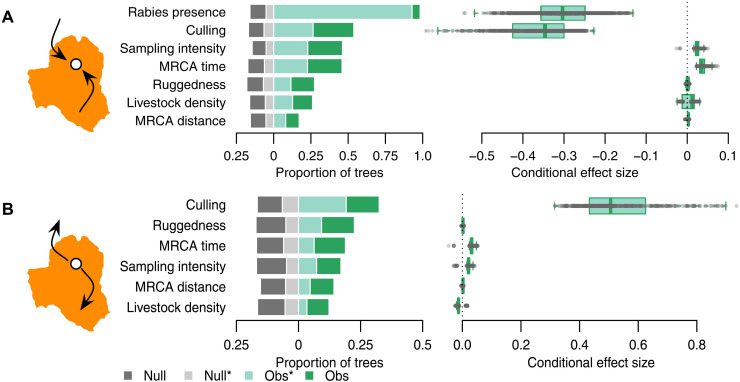
Effects of culling, landscape heterogeneity, sampling effort, and time on the velocity of rabies dispersal. Panels show predictors of viral dispersal events considering covariates calculated (**A**) at the destination of viral dispersals or (**B**) at the origin of viral dispersals, inferred from Bayesian phylogeography. Bar charts (left) show the proportion of 1000 trees that retained each effect and whether observed effects were statistically significant (Obs*, *P* < 0.05, light green) or retained but not statistically significant (Obs, *P* > 0.05, dark green). Gray bars show the expected frequency that each variable would be retained on the basis of a null model where true values were randomized for each tree and whether they were statistically significant (Null*, light gray) or not (Null, dark gray). Boxplots (right) show estimated effect sizes, conditional on being retained in the model. Gray points are observed effect sizes.

We next sought to test how culling affected the velocity of viral spread while controlling for phylogenetic uncertainty, temporal variation in viral spread rates, sampling effort, and other landscape-level variables. Specifically, we analyzed 1000 viral dispersal histories, each inferred from a randomly sampled posterior tree from the BEAST analysis and used the velocity inferred along each branch of each tree as a response variable in statistical models. When considering the landscape characteristics at the destination of viral dispersals, culling was retained in 53.8% of trees and, on average, slowed the velocity of viral invasions by 1.04 km/year [Fig. 4A; median/mean culled, 4.114/11.56 km/year (95% HPD, 0.01 to 47.86), versus non-culled, 5.16/13.8 (0.01 to 54.42)]. Reports of nearby rabies circulation during the previous 6 months also had a consistent (retained in 98.3% of trees) negative effect on viral dispersal speed, which may reflect the accumulation of immunity in bat populations from immunizing exposures or more intense culling in areas with recent spillover to livestock (*29*). We also observed a small positive effect of the time since the epizootic origin (“MRCA time”), indicating an acceleration of viral spread through time, and a separate effect signaling that heightened detection or reporting of cases (“sampling intensity”) improved detection of longer distance viral dispersals. The effects of culling were reversed when considering the landscape characteristics at the origin of viral dispersals, with culling accelerating viral spread by 2.41 km/year [Fig. 4B; median/mean culled, 7.4/19.24 (95% HPD, 0.02 to 78.16), versus median non-culled, 4.98/13.37 (95% HPD, 0.004 to 52.47 km/year)]. Although this effect was retained in only 32.5% of trees, it was the most consistently included effect and comparison with a null model composed by randomizing culling status showed that it would only be expected to be retained by chance in 18.0% of trees (Fig. 4B). The positive effect of culling is therefore unlikely to be an artefact of our model selection process. Together with the marginally positive neighbor culling effect identified by the ZIP SSM (Fig. 3), the opposite direction of culling effects at the origin and destination of viral dispersal events supports the conclusion that, while preventive culling may delay viral invasion, reactive culling is more likely to accelerate viral spatial spread.

## DISCUSSION

In Latin America, vampire bats have been culled for over 50 years with the hope that reducing bat population density will reduce rabies transmission within bats and thereby prevent human and livestock rabies mortality. We found that a large, geographically synchronized cull was associated with widespread reductions of vampire bat populations but had negligible effects on the occurrence or intensity of spillover to local livestock. Furthermore, although preventive culls stalled viral spread, culls in areas with active viral circulation had the opposite effect of accelerating the dissemination of rabies across the landscape. Collectively, our findings support the view that the long-term maintenance of rabies via spatial processes limits the efficacy of culling as it is now implemented and provide a rare empirical example of perturbation effects, where changes in reservoir host behavior following incomplete eradication compromise spillover management by promoting pathogen spatial spread.

Vampire bat culling policies reflect a presumed relationship between bat population density and rabies transmission and an implicit assumption that rabies is maintained locally within vampire bat populations, such that maintaining populations below a threshold could prevent spillover. Our findings are inconsistent with these core assumptions. Specifically, we showed evidence for rabies maintenance over large spatial scales with no periodicity (fig. S3) and a strong influence of VBR dynamics across neighboring areas (Fig. 3), which both argue against local maintenance. Furthermore, we found that reduction of vampire bat population densities following a large-scale culling program, as seen by the reduction in bat biting rates, failed to eliminate (Fig. 1B) or generate a corresponding reduction in rabies spillover to livestock (Fig. 2). This suggests that rabies incidence in bats was unaffected by culling. To our knowledge, this result is the first empirical test of whether culling bats reduces the spillover of any pathogen and therefore sets a valuable precedent given the role of bats as a source of viral zoonoses and the growing interest in preventing viral emergence. The lack of reduction in rabies spillover observed here aligns with a previous study that found that vampire bat colony size was not correlated with rabies seroprevalence, suggesting weak relationships between bat density and rabies incidence (*16*). Our results further suggest that the apparent absence of density-dependent transmission in a gregarious bat host arises from spatially mediated maintenance, perhaps in combination with nonlinear scaling of bite rates with bat density, as observed in canine rabies (*30*). Because rabies is only ephemerally present in individual bat colonies and can spread in low bat densities due to virus-induced behavioral changes that facilitate biting, population size is disconnected from incidence and no population thresholds exist under which rabies may be guaranteed not to invade. This disconnection may explain why even the spatially synchronized culling effort that we analyzed was insufficient to eliminate rabies from either the culled or non-culled districts in our study area and why the L1 virus appeared capable of spreading through culled and L3 enzootic areas, despite a presumably diminished supply of susceptible bats (fig. S4).

Our study also identified scenarios under which culling vampire bats could negatively affect human and animal health. Phylogenomic analyses showed that, while preventive culls might stall viral spread, the more widely practiced reactive culls appeared to accelerate viral spread (Fig. 4). The weak but slightly positive effect of culling on the intensity of spillover in neighboring districts in our ZIP SSM lends independent support to this unintended consequence of reactive culling (Fig. 2). Accelerating the arrival and possible establishment of enzootic rabies in bat populations increases human rabies risk directly from bat depredation on humans or handling of moribund rabid bats and indirectly through handling potentially infectious livestock. We hypothesize that accelerated viral spread results from heightened dispersal of infected survivors of culls. This behavioral response is enabled by the high sensitivity of vampire bats to disturbance (vampire bats often temporarily abandon their roosts following capture), combined with the prolonged incubation period of rabies, lasting weeks to months (*31*). Our results therefore support the hypothesis that culling wildlife may exacerbate disease spread by perturbing animal behavior or demography (i.e., “perturbation effects”). Perturbation effects have been predicted in theoretical and conceptual models of a variety of host-pathogen systems but typically have not been confronted with data from real-world culls (*1*, *32*–*36*). Empirical evidence therefore remains exceptional (e.g., badgers and tuberculosis in the United Kingdom) and has required large-scale experiments combined with labor intensive animal tracking data and/or indirect inference from incidence in spillover hosts (*37*, *38*). Incorporating genomic data into evaluations of human-wildlife interactions is another emerging frontier, as demonstrated by a recent study of the effects of hunting puma on the transmission of feline immunodeficiency virus (*39*). Our results show that carrying out pathogen genomic sequencing alongside culling of wildlife reservoirs can directly estimate how interventions affect not only spillover incidence but also pathogen spatial spread. Given the increasing availability of pathogen genomic data, this approach could be applied more broadly to assess cryptic effects of interventions in wildlife disease systems.

In the absence of alternative strategies to manage vampire bat populations or interrupt rabies transmission, culls will necessarily continue to limit vampire bat depredation on livestock and humans. Our findings show that the epidemiological context of culls influences whether they are beneficial, ineffective, or counterproductive. Specifically, our results suggest that reactive culling should be avoided because it does not reliably reduce local spillover incidence (Fig. 3) and may increase viral emigration (Fig. 4B). On the other hand, the decelerating effect of culls at the destinations of viral dispersals suggests that preventive culls may delay viral incursions. The dependence of culling efficacy on VBR spatial dynamics highlights the necessity of managing VBR as a regionally enzootic but locally epizootic infection, whereby spatiotemporally explicit knowledge of transmission dynamics in the bat reservoir guides decisions on the timing and location of culls. A preventive approach is already feasible where strong geographic barriers to bat dispersal enable forecasting of the route and velocity of VBR spread (*20*, *27*). Preventive interventions are a greater challenge in VBR-enzootic areas where the locations of future outbreaks are less predictable; however, network models of rabies spread among bat colonies offer a promising way forward (*40*). Reproductive suppression and self-disseminating vaccines targeting vampire bats are emerging tools to manage both rabies and bat populations, which might diminish counterproductive effects of disturbance-induced dispersal on rabies spatial spread (*41*–*43*). Moreover, the disturbance-induced dispersal of bats following capture observed here might be exploited to benefit rabies prevention by promoting vaccine spread to nearby populations. Further development of these tools would also alleviate animal welfare and bat conservation concerns associated with vampiricide, including the possibility of sublethal doses and exposures to non–vampire bat species (*13*).

Vaccination of livestock is a cornerstone for prevention of VBR but, unexpectedly, was not retained in our ZIP SSM, outwardly suggesting a negligible ability of vaccination to prevent rabies in livestock. Because rabies vaccine failures at the individual level are expected to be rare, we suspect that the inability of our model to detect a protective effect of vaccination may reflect pervasive reactive vaccination (i.e., vaccination that is intensified after local detections of rabies) in the region (*12*, *24*). Specifically, except for the first month that rabies was reported in a district, our 12-month cumulative measure of vaccination would have described a mixture of preventive vaccination, which would be expected to be negatively correlated with outbreaks and reactive vaccination, which is intrinsically positively associated with outbreaks. Unfortunately, shorter windows of vaccination that might disentangle these effects are biologically inappropriate because of the expected 12-month duration of vaccine-derived immunity. It is also possible that vaccination coverage was consistently too low to have detectable preventive effects and that the interval between successive outbreaks was longer than the duration of protective immunity, making reactive vaccination unable to yield a future preventive effect. The high burden of rabies in Peru despite bat culling and vaccination reinforces the need to increase livestock vaccination. Unfortunately, the unpredictability of risk that arises from noncyclical enzootic maintenance shown here (fig. S3) represents a barrier that will require research at the interface of epidemiology and the social sciences to incentivize intervention before outbreaks begin.

Our study shows how combining statistical and phylogenomic inference can reveal detailed epidemiological impacts of wildlife culling, even when data arise from uncontrolled interventions. Nevertheless, we faced several limitations. First, our sequence data extended until 2016 (the final year of the cull), but samples were unavailable for sequencing in subsequent years despite the continued circulation of VBR (Fig. 1). This means that additional dispersals out of culled areas could not be included in our phylogeographic analyses; however, there is no reason to expect that these dispersals would systematically differ from those observed during the majority of the culling period. Furthermore, the neighbor culling effect in the ZIP SSM (which included data through 2019) provides an independent validation of the spatial acceleration effect observed in our phylogeographic model, suggesting that additional sequencing would be likely to strengthen this counterproductive effect of culling. Second, although we observed patterns consistent with geographically widespread reductions of vampire bat populations, the magnitude of these reductions was modest (Fig. 2). We speculate that this might reflect rapid recolonization of roosts after culling (i.e., “vacuum” effects), low efficacy of the vampiricide product used, or that bites on livestock, although correlated with vampire bat population density, are an imperfect proxy (*23*). Ultimately, targeted field studies will be useful to understand how bat population dynamics and dispersal respond to culls and whether the epidemiological contexts under which culling might be effective are operationally and ethically achievable.

In conclusion, the context-dependent effects of culling wildlife for disease control have been extensively discussed in conceptual and theoretical models but rarely empirically tested. Our study demonstrates that analyzing epidemiological and genomic data alongside real-world culls can provide fundamental insights into the determinants of viral maintenance within wildlife reservoirs while resolving how ecological and behavioral responses of wildlife to culling translate into pathogen spillover risk and spatial spread. Our results provide a rare glimpse into the benefits and risks of culling for vampire bat rabies prevention and suggest that bat culls carried out for decades by most countries in the Americas may have had limited benefits for preventing lethal rabies infections in humans or livestock, despite reducing vampire bat bite rates. Epidemiologically aware culls could be more effective but remain difficult to implement and are unlikely to be applied at a scale that would be sufficient to eliminate VBR. These findings incentivize the development of strategies to anticipate and interrupt VBR transmission and spillover.

## MATERIALS AND METHODS

### Experimental design

We analyzed a variety of epidemiological and genomic datasets collected through the active and passive surveillance activities of regional and national authorities in Peru. We focused on an area of southern Peru characterized by particularly high rabies incidence and where a large-scale campaign was carried out by the regional government in efforts to reduce rabies incidence in domestic livestock. Data sources, laboratory analyses (where relevant), and statistical approaches are described in detail below.

### Livestock rabies data

The monthly number of rabies outbreaks in livestock and the monthly number of livestock vaccinated against rabies in the study area from 2003 to October 2019 (200 months) were provided by the National Service of Agrarian Health of Peru (SENASA). Briefly, SENASA responds to reports of animal morbidity and mortality from livestock owners or local veterinarians. When clinical signs are consistent with rabies (e.g., ataxia, hind limb paralysis, and seizure), a brain sample is collected and tested for rabies using the fluorescent antibody test at the national headquarters in Lima, Peru. Over the course of our study, SENASA implemented no major changes in rabies surveillance, and an earlier analysis of non-rabies pathogens in the same livestock populations found no systematic increase in animal health surveillance effort since an initial rise, coinciding with the implementation of the current surveillance system (2003 to 2008) (*27*). Our dataset described the date and GPS locations of 1029 outbreaks in the AAC area. Three outbreaks from northern Cusco (see Fig. 1A) were excluded because of the prior knowledge that these were caused by a distinct lineage of vampire bat rabies that was believed not to circulate elsewhere in AAC (*20*). Brains from laboratory confirmed rabies positive livestock were provided to this study for sequencing (data S1). SENASA also recorded the characteristics of farms that had suspected or confirmed rabies outbreaks. Most AAC properties with livestock were family farms (76%), with mixed production of meat and dairy and a small number of animals (median, 11 animals; SD = 33.2). Farms with exclusive production of meat or dairy were rare, 6% and 0.6%, respectively, and showed no clear changes in frequency by year (binomial GLM, *P* > 0.05 for both).

### Bat bite questionnaires

To approximate changes in bat abundance throughout the culling period, 14 technicians from the culling program recorded the number of domestic livestock with visible evidence of vampire bat bites. Bites are easily recognized by their size, shape, and prolonged bleeding from anticoagulants in bat saliva. Each technician was trained at the beginning of the project to identify bat bites, to use GPS to record property locations, and to record animal bite data. Technicians were assigned a fixed number of districts and a target number of properties to survey monthly. Paper records of data were reviewed and validated by SENASA and were digitized for this study. During visits, technicians actively counted the number of bitten animals with assistance from property owners. This effort varied across the 33 evaluated districts [mean, 22 (range, 1 to 91) properties evaluated per district per month; mean, 310 (range, 6 to 1491) individual animals evaluated per district per month]. Given the small number of animals at farms in our study area (see above), it was generally possible to inspect all animals for bites.

### Culling data

All culling activities were carried out by the Regional Government of Apurimac under the authority of regional resolution number 368-2018 GR.APURIMAC.GR. For each cull, bats were captured by setting mist nets (6 or 12 m by 2.6 m) outside of known vampire bat roosts (25.9% of capture events) or at properties where bats were reported to have recently attacked livestock (74.1% of capture events, fig. S6). For captures at foraging locations, nets were installed at ground level at 6:00 p.m. and opened from the time of darkness until dawn. The number and size of nets used varied according to the orientation and accessibility of livestock corrals. Captures avoided nights around the full moon and nights with rain or heavy winds. Captures at roosts were carried out diurnally with bats encouraged to leave roosts by entering roosts with butterfly nets or using smoke. Bats were captured in nets placed at roost exits. Culling events generally lasted 1 day or night per location. In all cases, culls applied vampiricide poison (Diphenadione) to captured bats that were released at the site of capture. Data were aggregated and analyzed in the SSM as the number of bats that were treated with vampiricide per district per month (data S3).

### Statistical analysis of bat bites on livestock

To quantify the impact of culling on bat population size, we investigated the potential reduction of bites during the culling period as proxy. We fitted a binomial GLMM with logit link function to the number of animals bitten in a farm and the number of animals evaluated, with district used as a random effect. A second binomial GLM used whether farms had reported at least one bitten animal (0 or 1) as response variable. Both models were fitted with continuous month as the independent variable. Models fitted well (Fig. 2) and showed no scaled residual dispersal or deviation [evaluated in R package DHARMa; (*44*)]. A GLM was used for the farm level model rather than a GLMM because the “district” random effect increased dispersion in the residuals.

### Bayesian zero-inflated Poisson state-space model

We used a Bayesian ZIP model under a state-space framework to investigate the impact of culling on local rabies incidence and on viral spread between districts. The SSM included an observation process that allowed us to account for district-specific underreporting of rabies outbreaks and a biological process that characterized rabies outbreaks in livestock through the ZIP. The ZIP was chosen because the outbreak count data exhibited overdispersion and excess zeros (94.7% zeros across all districts). This modeling approach allowed us to separate effects on the occurrence of outbreaks in a district from the number of outbreaks in each district. Briefly, we modeled the occurrence of outbreaks in a district as a function of spatial variables because rabies outbreaks are initiated by larger-scale spatial processes such as invasion from neighboring districts. Furthermore, culls might have spatially pervasive effects on rabies dynamics due to either a homogenizing effect of bat dispersal or the influence of culls on bat dispersal (neighbors culling). In contrast, the number of outbreaks in each district was assumed to be generated by local epidemiological processes (here defined by AR processes in livestock rabies incidence and a trend over time) and human interventions (livestock vaccination effort and metrics of local bat culling). To confirm suitability of our ZIP model compared to a simpler model structure, we also fit a standard Poisson SSM. The correlation between observed and estimated values from the Poisson SSM was 79% compared to 91% from the ZIP SSM, indicating improved fit in the ZIP SSM.

#### Biological process

The unobserved number of real outbreaks in each district *i* and month *t* was modeled as a two-component mixture model. Formally, the probability mass function for the ZIP model was defined as

$$\mathrm{Pr}(Y_{i,t}=0)=z_{i,t}+(1-z_{i,t})e^{-r_{i,t}}$$

$$\mathrm{Pr}(Y_{i,t}=y_{i,t})=(1-z_{i,t})\frac{({r_{i,t}}^{y_{i,t}})e^{-r_{i,t}}}{y_{i,t}!}$$

where *z*_*i*,*t*_ is the proportion of extra zeroes in the process (as might be determined by neighborhood effects of the focal region *i* at time *t*) and *r*_*i*,*t*_ is the Poisson rate describing the count of outbreaks for those regions and times whose neighborhoods encourage the occurrence of rabies. In practice, this is implemented as a hurdle model by first determining whether a rabies outbreak is feasible via a Bernoulli distribution *z*_*i*,*t*_ ~ Bernoulli(1 − *z*_*i*,*t*_) where 1 − *z*_*i*,*t*_ = ϕ_*i*,*t*_ and then modeling the number of outbreaks from a Poisson distribution *O_i,t_* ~ Poisson(*r*_*i*,*t*_). It follows that the estimated number of outbreaks *y*_*i*,*t*_ = *z*_*i*,*t*_*O*_*i*,*t*_ with

$$\mathrm{logit}(\varphi_{i,t})=\theta_{0}+\theta_{1}{\mathrm{neighbors}}_{i,t-1}+\theta_{2}{\mathrm{neighbors}}_{i,t}+\theta_{3}\mathrm{culled}\_{\mathrm{neighbors}}_{i,t-1}$$

The intercept θ_0_ corresponds to the baseline proportion of zero outbreaks, and the parameters θ_1_ and θ_2_ correspond to the coefficients governing the effect of presence of rabies in the neighboring districts at *t* and *t −* 1, respectively. Rabies is considered present if at least one of the adjacent districts has (or had) rabies, otherwise is absent. This covariate serves to inform if rabies outbreaks are more likely to occur if a neighboring district now has or had rabies in the previous month. These neighborhood effects generate the apparent zero inflation in the data. Similarly, θ_3_ corresponds to the coefficient governing the effect of culling (cumulative number of bats culled) in the neighboring districts at *t −* 1. Culling in the neighboring districts could increase rabies spillover in the focal district through immigration of displaced, infected bats or could decrease rabies if reductions in incidence by culling in neighboring areas reduced opportunities for spread into focal districts. This time lag was chosen with consideration of the incubation periods of rabies: on average, 19 days in vampire bats and 17 days in cattle (*31*). As such, livestock in neighboring districts could be exposed from day 0 (in the case that an infectious bat disperses on the day of culling) until day 19 (if an exposed bat disperses at the start of the incubation period), meaning rabies cases arising from bat dispersal might be expected to occur between day 17 and day 36 (i.e., approximately 1 month). However, we also considered a 2-month lag that had a higher Deviance Information Criterion (ΔDIC, 180).

The number of outbreaks *O*_*i*,*t*_ was characterized by local processes defined through a function of covariates to capture the overall disease dynamics and the local factors influencing those dynamics

$$\mathrm{logit}(O_{i,t})=\beta_{0,i}+\beta_{1}t+\beta_{2}\mathrm{rabies}\_{\mathrm{outbreaks}}_{i,t-1}+\beta_{3}\mathrm{rabies}\_{\mathrm{outbreaks}}_{i,t-2}+\beta_{4}{\mathrm{culling}}_{i,t-1}+\beta_{5}{\mathrm{vaccination}}_{i,t}$$

The intercept β_0,*i*_ corresponds to the district-specific baseline number of rabies occurrences. The parameter β_1_ corresponds to the linear trend on time across all districts, and the AR parameters β_2_ and β_3_ correspond to the coefficients governing the effect of rabies outbreaks at *t −* 1 and *t −* 2, respectively, and serve to emulate the disease dynamics over time (*18*, *25*). We note that this AR uses the rabies outbreak data as an autocovariate instead of the expected number of outbreaks so that the count process is also directly informed by the data. This considerably improved model fit. The parameter β_4_ corresponds to the coefficient governing culling. Because the time frame at which culling might affect local rabies dynamics was unclear at the start of our analysis, we created three culling covariates comprising the cumulative number of bats to which vampiricide was applied in the focal district during the past (i) 3, (ii) 6, or (iii) 12 months. However, because these covariates are not independent (e.g., i and ii are nested within iii), instead of adding three separate covariates, we compared the goodness of fit and DIC of three distinct models to determine the most appropriate culling window. We note that these time lags were not explored for the zero process because dispersal to neighboring districts from culling is thought to be more immediate, as mentioned above. We further compared models without either or both culling covariates. Last, we compared models with and without the remaining parameter β_5_ that corresponds to the coefficient governing the impact of the average vaccination effort across the previous 12 months on the number of rabies outbreaks. A sliding window of 12 months was chosen for this covariate because rabies vaccination is recommended annually for livestock in Peru (*24*). Table S1 summarizes these models and the resultant ΔDIC.

#### Observation process

The observed number of outbreaks in each district and month, *Y_i,t_*, was modeled as a binomial distribution with probability of detection in each district, *q*_i_, and the number of trials set to the latent number of outbreaks, *y_i,t_*. The prior for *q* was defined by a beta distribution with the mean set as the proportion of underreporting for each district previously estimated using questionnaire data in (*24*) and variance of 0.01.

#### Priors and model fit

Unless specified above, the remaining coefficients were drawn from Gaussian priors with mean of 0 and variance of 100. The exception was the two neighborhood parameters in the zero-inflated process that were given an exponential prior distribution with mean of 0.5 to preserve their positivity. The models were fit in JAGS 4.3 [using R package “rjags”; (*45*)] for 50,000 iterations with the first 25,000 interactions discarded as burn-in and retaining every 10th iteration. Convergence across three independent chains was assessed through visual inspection of the posterior chains and a Gelman-Rubin diagnostic test [in R package “coda”; (*46*)].

### Multiplex whole genome amplification, library preparation, and sequencing of rabies viruses

RNA was extracted from brain samples collected between 2013 and 2016 using TRIzol, according to the manufacturer’s instructions and was shipped on dry ice to the MRC-University of Glasgow Centre for Virus Research in the United Kingdom. Whole genome sequencing was also carried out using banked RNA extracted from a subset of older samples (1997 to 2012) for which N and/or G-L intergenic regions were previously described (*20*).

Multiplex primers were constructed with primalscheme (*44*) using complete VBR genomes from South America available on GenBank (accession numbers AB519642, KM594041, and EU293113) and “hybrid” genomes generated by swapping out the *nucleoprotein* gene from public genomes for representative *nucleoprotein* gene sequences from each circulating rabies lineage in Peru. This was necessary because complete genomes from Peruvian VBRs were unavailable at the start of our project. A 400–base pair (bp) amplicon length with a 40-bp overlap setting generated 37 overlapping primer regions. These primers were tested over two small initial sequencing runs using various samples from different Peruvian viruses, and 25 primers were adapted or redesigned around regions of low coverage. The final multiplex polymerase chain reactions (PCRs) had a total of 91 primers, comprising 50 primers in pool A and 41 primers in pool B of varying amounts (data S2).

The multiplex whole genome amplification protocol used was a combination of protocols from Brunker *et al.* (*47*) and Quick *et al.* (*48*). Briefly, complementary DNA (cDNA) was generated using the ProtoScript II First Strand cDNA Synthesis Kit [New England Biolabs (NEB)]. Multiplex PCR followed using 2.5 μl of cDNA, 1.5 μl of either primer pool A or B, and Q5 Hot Start High-Fidelity DNA Polymerase (NEB), cycled with the following conditions: initial denaturation at 98°C for 30 s, followed by 45 cycles of denaturation at 98°C (15 s) and 65°C (5 min) combined annealing and extension, and final extension at 65°C for 15 min. Amplification was visualized on 1.5% agarose gels for 400-bp PCR products. PCR products from the same sample were combined, purified using a QIAquick PCR purification kit (Qiagen), and quantified using a Qubit dsDNA HS assay kit (Thermo Fisher Scientific).

Library preparation was performed with a KAPA LTP Library Preparation kit (Roche) using the NEBNext Adaptor for Illumina (NEB) and NEBNext Multiplex Oligos for Illumina (Dual Index Primers Set 1; NEB) according to manufacturer’s instructions. Sample libraries were checked for quality and sizing on the 4200 TapeStation System (Agilent Technologies). Libraries were pooled, adjusted, and sequenced using the Illumina MiSeq system on the MiSeq Reagent Kit v2 (500 cycles) sequencing cartridge with paired end reads of a 250-bp length. Raw Illumina sequencing reads were adapter trimmed, quality-filtered, aligned with Burrows-Wheeler Aligner using Maximal Exact Matches (BWA-MEM), and primer-clipped with iVar (*49*, *50*). Consensus full genome sequences were generated using the following nucleotide calling rules: Ambiguity nucleotide codes were applied if two bases were present above 25% coverage, and sites with coverage less than 5 reads were designated N.

### Phylogenetic analyses

#### Nucleoprotein gene analysis

To classify viral lineages circulating in AAC, a phylogenetic analysis was carried out using 319 sequences generated here, along with representative VBR *nucleoprotein *gene sequences available from GenBank (*n* = 316). Sequences were aligned in Multiple Alignment using Fast Fourier Transform (MAFFT) and analyzed in BEAST v.1.10.4 using the Bayesian skyline demographic model, the relaxed lognormal molecular clock, and separate General Time Reversible (GTR) + gamma substitution models for codon positions 1 + 2 versus codon position 3. Tip dates were converted to decimal form where information on the month and day of collection was available. When only collection year was available (primarily for reference sequences), we assigned 1 year of uncertainty in the collection date.

#### Whole genome sequences

Phylogeographic analyses were carried out on whole genome sequences of L3 viruses detected in AAC between January 1997 and January 2016. This analysis excluded sequences with missing spatial or temporal metadata (*n* = 6), sequences that comprised a clade of long branches basal to the remaining monophyletic clade of L3 sequences (*n* = 5), and five sequences with possible sequencing and/or metadata errors (residuals of relationship between temporal and evolutionary divergence from the estimated root > 0.01), creating a final alignment of 290 rabies virus genomes (data S1). TempEst v.1.5.3 analysis of a maximum likelihood tree built from this alignment in iqTree found strong evidence of clock-like evolution (slope = 3.16 × 10^−4^, *R*^2^[coefficient of determination] = 0.62). Analyses in BEAST used the Bayesian skyride demographic model, the relaxed lognormal molecular clock, and the Cauchy-distributed continuous phylogeographic model, which was favored over gamma and lognormal models (Bayes factor, 36.8 and 52.8, respectively) (*51*, *52*). Genomes were partitioned into coding and noncoding regions, which were concatenated into two alignments. The coding sequence alignment was further partitioned by codon position, grouping positions 1 and 2 separately from codon position 3. All BEAST analyses were carried out in duplicate for 150 million generations each, with trees and parameters sampled every 15,000 generations. Convergence within and between runs was confirmed in Tracer.

### Evolutionary analysis of drivers of viral dispersal

We used the posterior set of spatiotemporally annotated phylogenies from BEAST to analyze how culling, along with other environmental and spatiotemporal factors, affected the speed of viral spatial spread. We first used the R package Seraphim to extract the inferred locations and times of each branch in each of 1000 randomly sampled, post–burn-in trees (*53*). We then used the distance traveled and time-elapsed to infer branch specific velocities. Existing software to evaluate the environmental effects on phylogenetic branch velocities is now unable to accommodate explanatory variables that vary throughout the history of the tree (e.g., variable culling effort through time) or to separate effects at the origin versus destination of dispersal events. To identify both spatial and temporal correlates of branch specific velocities while accounting for phylogenetic uncertainty, we therefore fit GLMs to the spread history inferred from each posterior tree and summarized the frequency that each variable was retained by automatic reverse model selection (stepAIC in R) and the effect sizes of each variable, conditional on being retained by model selection. As a control, we repeated this process after randomizing one explanatory at a time to calculate the frequency that each variable might be retained by our model selection procedure by chance. Because some short branches had biologically implausible velocities, we excluded the top 1% of estimated velocities. We also limited the analysis to branches that originated after 2003 for consistency with epidemiological data.

To test the effects on viral immigration and emigration, we fit separate models using landscape characteristics at the (i) origin and (ii) destination of the reconstructed viral dispersal events. In the context of culling, “origin” effects would describe the consequences of culls necessarily carried out in areas with preexisting viral circulation (i.e., reactive culling), and “destination” effects described the effects of culls carried out before the arrival of a specific genetic lineage (i.e., preventive culling with respect to the invading lineage). Assessing the effects of culls on arrivals into genuinely rabies-free areas was challenging given the uncertainty in spatiotemporal scale to define the absence of rabies. Nevertheless, we retained dispersals into areas with recent reports of rabies in our destination models because we were interested in how patterns of bat and virus dispersal could influence the dynamics of viral spread into culled areas, regardless of whether other strains of rabies were present. We measured culling at the district level as a spatiotemporally explicit binary variable. Additional explanatory variables hypothesized to affect viral dispersal velocity included livestock density (sum of cows, sheep, goats, and pigs from Food and Agriculture Organization’s Gridded Livestock of the World) and landscape ruggedness (“tri” in the terrain function of the raster package in R), each calculated within a 10-km radius of each inferred branch origin or destination. To investigate systematic changes in viral velocity during the epizootic, we also included the temporal and geographic distance between each branch origin and the inferred MRCA of its associated tree. Because greater local sampling intensity might facilitate detection of rare long distance viral dispersals, we included the average number of suspected or confirmed rabies outbreaks per year within 10 km of each branch origin or destination during the years when rabies was locally present as a proxy for sampling intensity. The destination model also included a binary variable tracking the presence of rabies cases within 5 km during the previous 6 months. The 6-month window was selected to capture the possibilities that recent outbreaks triggered culls or that recent viral circulation increased immunity in the local bat population, as well as to minimize overlap with the sampling intensity variable. Approximately 54% of viral dispersals arrived in areas without recent evidence of viral circulation. The presence of rabies was not included in the origin model because rabies was present by definition. Vaccination of livestock was excluded from the analysis of viral spatial spread because successful viral dispersals (i.e., those with potential to result in onward transmission) are realized exclusively by bats. In other words, livestock represent a convenience sample of the viruses circulating in local bat populations but are assumed not to contribute to viral spatial spread.

## Supplementary Materials

### This PDF file includes:

Supplementary Text Figs. S1 to S6 Table S1 Legends for data S1 to S3 Code S1 to S3
