# Supplementary material for: Effects of culling vampire bats on the spatial spread and spillover of rabies virus
Source: Sci Adv. 2023 Mar 10;9(10):eadd7437. doi: 10.1126/sciadv.add7437 (PMC10005164; doi:10.1126/sciadv.add7437)
Supplement: Supplementary file 1 — Supplementary Text Figs. S1 to S6 Table S1 Legends for data S1 to S3 Code S1 to S3 [file sciadv.add7437_sm.pdf]

Science Advances
AAAS

Supplementary Materials for

Effects of culling vampire bats on the spatial spread and spillover of rabies virus

Mafalda Viana *et al.*

Corresponding author: Daniel G. Streicker, daniel.streicker@glasgow.ac.uk

Sci. Adv. **9**, eadd7437 (2023)
DOI: 10.1126/sciadv.add7437

The PDF file includes:

Supplementary Text
Figs. S1 to S6
Table S1
Codes S1 to S3
Legends for data S1 to S3

Other Supplementary Material for this manuscript includes the following:

Data S1 to S3

Supplementary Text

Performance and supplementary results for Bayesian ZIP SSM

Model selection

The most biologically appropriate timescale for potential effects of culling on rabies dynamics was unknown at the start of our study and if mis-specified could have led to incorrect conclusions on the value of these interventions for preventing rabies spillover. For example, removal of bats that were actively infected by or incubating rabies at the time of culls might lead to rapid reductions in livestock rabies. In contrast, longer term effects might arise if culls reduced incidence at the population level by depleting susceptibles. We therefore compared the fit of models using a range of time windows (3, 6 and 12 months) using DIC. We found that local effects of bat culling were greatest (though still weak) at 6 months prior to a focal date (Table S1). We also found that neighbor culling effects were greater at 1 month prior than at 2 months prior, supporting the hypothesis that short term behavioral responses to culls could facilitate viral spatial spread.

Royama triangle

The posterior distributions of the AR parameters can be used to form a Royama triangle (Fig S3), useful to investigate the dynamics of viral maintenance and periodicity across the AAC *(25, 26)*. This revealed that rabies is persistent across the study area but not predictably cyclic, suggesting asynchronous outbreaks, as expected under a metapopulation model of VBR maintenance *(15)*.

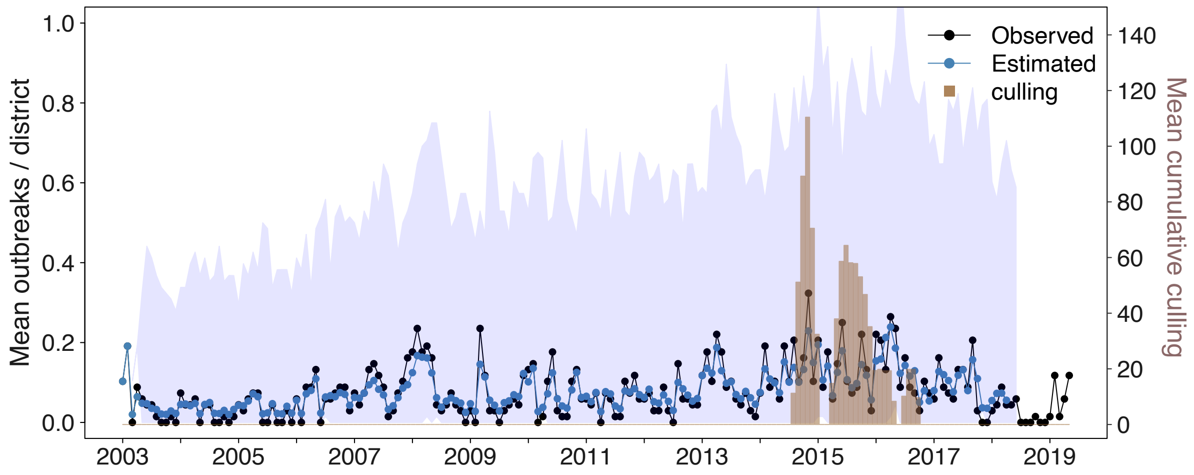

Fig. S1.
Performance of the Bayesian ZIP SSM. Mean observed (black) and estimated (blue; with shaded 95% credible intervals) outbreaks across all districts over time. Brown bars correspond to the cumulative mean number of bats culled.

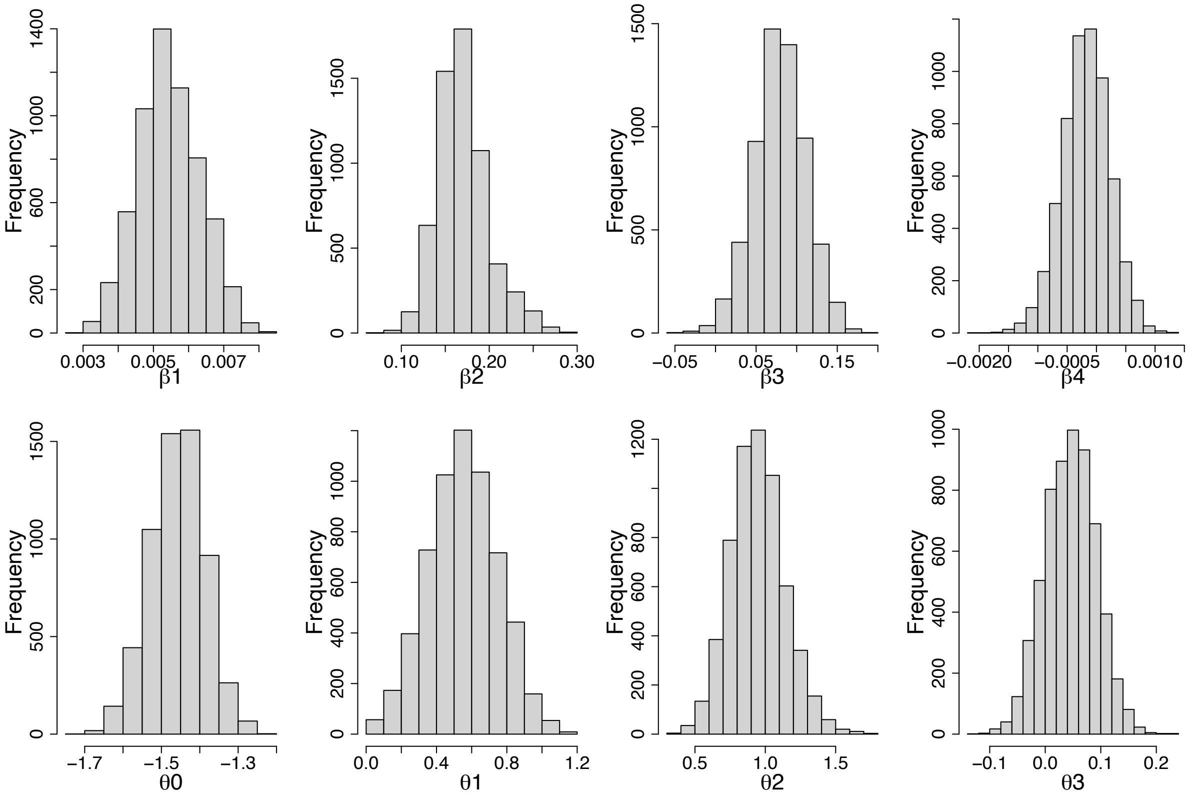

Fig. S2.

Histograms of the posterior distributions of the main parameters in the Bayesian ZIP SSM model. Notation as in equations 1 and 2 in main text. The prior for all these distributions was uninformative and drawn from a normal distribution with mean 0 and variance 100 with exception of θ1 and θ2 which were drawn from an exponential distribution with mean 0.5.

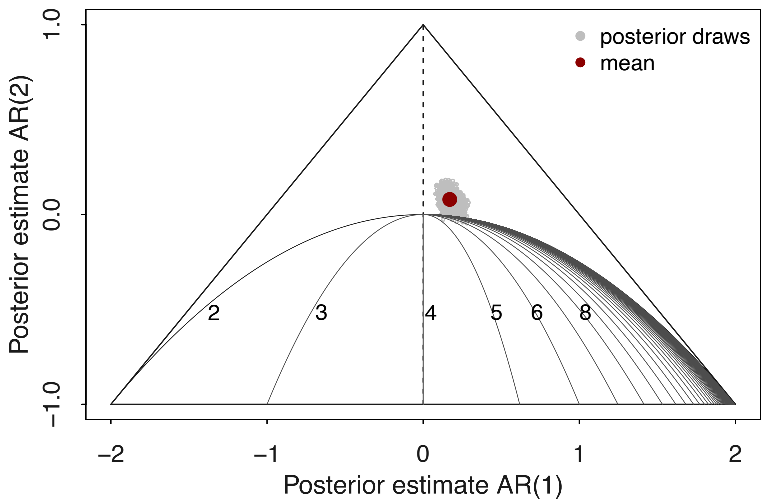

Fig. S3.
Royama triangle showing enzootic but non-periodic rabies outbreaks across all districts. The triangle is composed of the posterior distributions of the first and second order autoregressive components of the ZIP model. Parameter combinations outside the triangle lead to extinction, conversely, inside indicates enzootic maintenance. Below the semicircle the dynamics are cyclic and above they are stable. Above to left of the dashed line populations exhibit direct density-dependence.

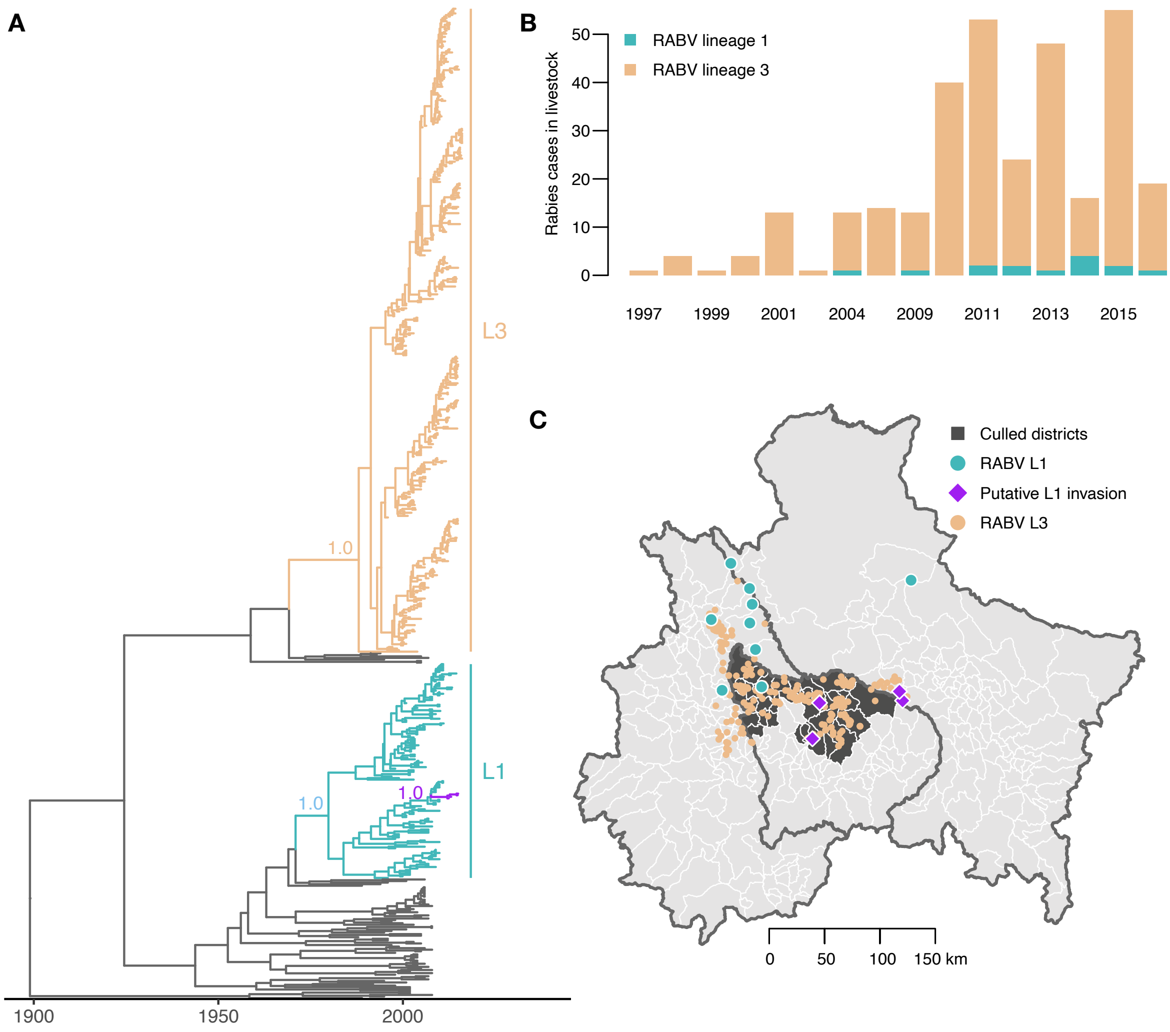

Fig. S4.
Rabies virus lineages detected in Apurimac, Ayacucho, and Cusco ('AAC', 1997-2016). (A) Bayesian phylogenetic tree of newly generated and reference sequences identifies two previously described viral lineages in AAC. Viral lineages detected outside of AAC (including from other countries) are shown in grey. This analysis revealed two lineages circulating in our study area during the culling period, with one lineage (L1) appearing to have invaded the study area recently. (B) Time series of the distribution of 319 sequenced viruses by viral lineage. (C) Map of AAC with the locations of viral lineages. Purple diamonds show the monophyletic clade of L1 viruses within AAC (see panel A).

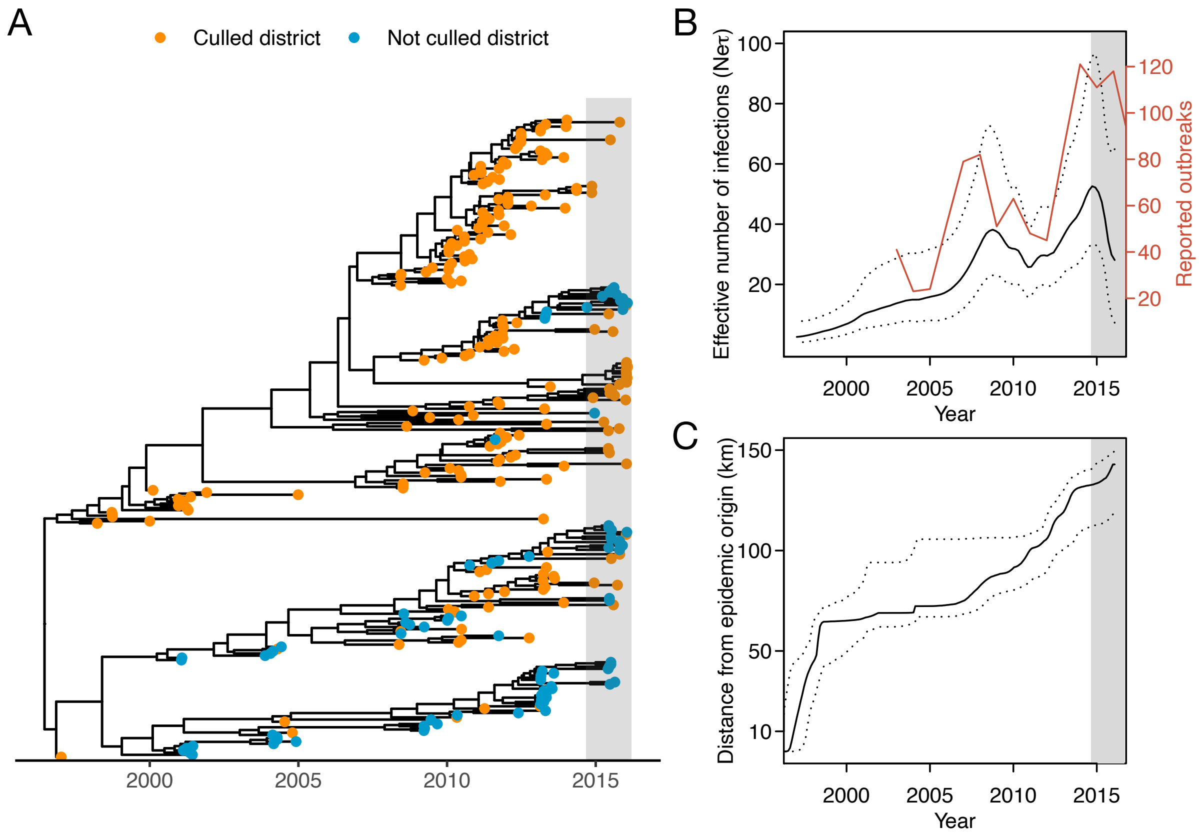

Fig. S5.
Evolutionary relationships and inferred demography of rabies virus lineage 3 in AAC. (A) Rabies virus infections estimated from genomic and case data. Median effective number of RABV infections (thick black line) estimated using a Bayesian skyride model, representing the produce of effective population size (Ne) and generation time (tau). Dashed lines represent 95% highest posterior density (HPD) intervals. Red line shows the number of outbreaks reported to the Peruvian national surveillance system in AAC during the same time period. Grey shading indicates culling period. (B) Spatial expansion of RABV, depicted as the cumulative geographic distance from the inferred outbreak origin through time. Lines represent the median (solid) and 95%HPD (dashed) distance in kilometers (km). Inferred demographic patterns were consistent with observations from surveillance data and earlier studies showing viral expansions within this region of Peru *(20, 27)*. Specifically, we noted parallel fluctuations of the effective number of viral infections and reported outbreaks and gradual increases in the spatial area of AAC infected with rabies.

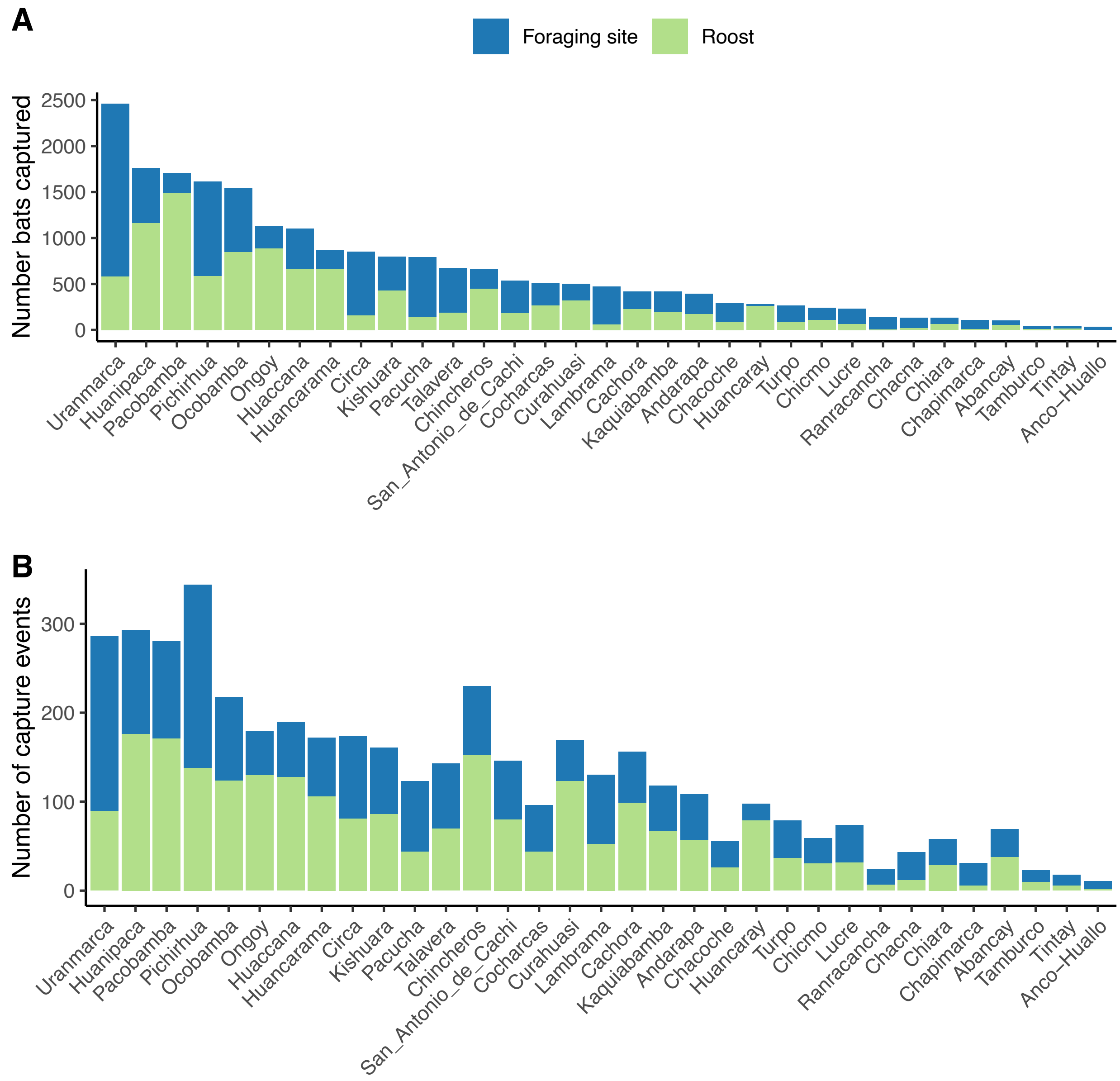

Fig. S6.
Bat capture and culling effort in 33 districts of Apurimac. (A) Number of individual bats captured and treated with vampiricide. (B) Number of capture events (i.e., nights of attempted vampire bat capture) per district. In both panels, captures at foraging sites are indicated in blue and captures at vampire bat roosts are indicated in green. Districts in panel B are arranged in matching order to panel A.

Model	DIC	ΔDIC
Base model	5048	199
Base model + Neighbor culling + Local culling window of:		
▪ 3 months	5289	440
▪ 6 months	**4849**	0
▪ 12 months	5205	356
*Base model + Local culling**	5299	450
Base model + Neighbor culling	5022	173
*Base model + Neighbor culling + Local culling** + Vaccination	5274	425
Base model + Vaccination	5549	700
Base model + Neighbor culling (t-2) + *Local culling**	5030	180

*Using local culling window of 6 months due to lowest DIC.

Table S1.
Comparison of alternative Bayesian ZIP SSMs. Model selection was performed on the base model of rabies outbreak size in livestock (as explained in equations 1-2) to identify the most parsimonious time window for the local cumulative culling and to explore the effects of culling and vaccination on rabies outbreaks.

Other Supplementary Materials for this manuscript include the following:

Data S1. Metadata for rabies positive livestock samples sequenced and included in phylogenetic analyses (https://zenodo.org/record/7457524/files/DataS1_NGSSamples.csv).
Data S2. Multiplex PCR primers for tiling amplicon generation (https://zenodo.org/record/7457524/files/DataS2_PCR_Primers.pdf).
Data S3. Vampire bat culling data at the month and district levels from Apurimac, Peru (https://zenodo.org/record/7457524/files/DataS3_culling_data.csv).
Code S1. JAGS code for Bayesian zero inflated Poisson state space model of livestock rabies occurrence and intensity (https://zenodo.org/record/7457524/files/CodeS1_JAGSmodel_ZIPculling.txt).
Code S2. BEAST XML file for continuous phylogeographic analysis of rabies virus genome sequences (https://zenodo.org/record/7457524/files/CodeS2_BEAST_cauchy.txt)
Code S3. R code for statistical analyses of viral dispersal speed (https://zenodo.org/record/7457524/files/CodeS3_phyloDispersal.R).
